# Efficacy of elongated needles for motor and balance function after a stroke: a systematic review and meta-analysis

**DOI:** 10.3389/fneur.2025.1550611

**Published:** 2025-05-30

**Authors:** Shuyan Zhang, Haichun Zhou

**Affiliations:** ^1^Heilongjiang University of Chinese Medicine, Harbin, China; ^2^The Fourth Affiliated Hospital of Heilongjiang University of Chinese Medicine, Harbin, China

**Keywords:** acupuncture, elongated needles, stroke, motor and balance function, systematic review, meta-analysis

## Abstract

**Introduction:**

This systematic review aimed to evaluate the efficacy and safety of elongated needle therapy in improving motor and balance functions after stroke, to inform its clinical adoption in rehabilitation.

**Methods and Analysis:**

We searched PubMed, Web of Science, EMBASE, Medicine, CNKI, CBM, Wanfang, and VIP from inception to May 1, 2024, for randomized controlled trials (RCTs) assessing elongated needles for post-stroke limb movement and balance dysfunction. Primary outcomes were balance and motor ability; secondary outcomes included Activities of Daily Living (ADL) and adverse events. Meta-analysis used RevMan 5.4 and Stata 16.0. Heterogeneity was explored via subgroup/meta-regression/sensitivity analyses (if significant). Two reviewers independently assessed bias risk using Cochrane tools. Outcome quality was evaluated with GRADE.

**Results:**

This meta-analysis included 18 randomized controlled trials (RCTs), encompassing a total of 1,230 subjects. The results indicated that elongated needles, utilized either as a solo intervention or in conjunction with other modalities, markedly enhanced balance capabilities in stroke patients when compared to conventional acupuncture, rehabilitation training, oral Chinese medicine, and alternative therapies including electroacupuncture and acupoint sticking (MD = 6.34, *P* < 0.001, *I*^2^ = 85%, 95% CI = 4.80–7.89). Furthermore, elongated needles, whether applied alone or in combination with other therapies, significantly improved the motor function of limbs in patients (SMD = 1.21, *P* < 0.00001, *I*^2^ = 92%, 95% CI = 0.63–1.79). Additionally, elongated needles, when used alone or in conjunction with other treatments, demonstrated greater efficacy in enhancing patients' activities of daily living compared to conventional acupuncture alone, rehabilitation training, or their combined regimen (SMD = 1.13, *P* < 0.00001, *I*^2^ = 83%, 95% CI = 0.70–1.55). However, further clinical research is warranted to substantiate the advantages of elongated needles over other therapies, including electroacupuncture and acupoint sticking. In terms of safety, the overwhelming majority of the studies included in the analysis reported the absence of adverse reactions.

**Conclusion:**

Evidence from current studies indicates that elongated needle may improve post-stroke patients' balance and motor function, and enhance their daily living skills. However, the number of rigorous scientific studies is limited, and there is considerable variability across studies, limiting the confidence in these findings. Therefore, the clinical effectiveness of this treatment still requires additional validation. It is imperative to conduct more high-quality, large-scale, multi-center RCTs that conform to international guidelines to establish the efficacy of this therapy's clinical applications.

**Systematic review registration:**

https://www.crd.york.ac.uk/prospero/, identifier CRD42024542151.

## 1 Introduction

Stroke is characterized by a disruption in cerebral blood circulation, resulting in localized neurological dysfunction. Classified into ischemic and hemorrhagic subtypes, stroke is the second most common cause of death and the leading cause of disability worldwide (1). In recent years, there has been an increase in the incidence and mortality of stroke, attributed to the progressive aging of the global population. Despite advancements in modern medicine and rehabilitation techniques enhancing survival rates, stroke survivors frequently endure various sequelae due to central nervous system damage. In severe instances, these complications can result in lifelong disabilities, which significantly impact the physical and psychological wellbeing of patients (2–4). A cohort study (5) shows that stroke survivors frequently encounter difficulties in performing daily activities as a result of complications like hemiplegia, depression, aphasia, and cognitive impairments. Therefore, tackling and rehabilitating stroke-related sequelae continue to be significant challenges within the medical field.

Balance is categorized into dynamic and static types and is influenced by various factors, including sensory input, central integration, and motor control (6, 7). Balance and motor function disorders are prevalent sequelae of stroke. Research (8) has demonstrated that 6 months post-discharge from hospitals or rehabilitation centers, the fall incidence rate among stroke survivors ranges from 50% to 70%, mainly due to impaired motor and balance capabilities. This not only severely impacts the daily activities of patients but also imposes considerable psychological and economic burdens on them and their families. Consequently, enhancing balance and motor functions and preventing falls are critical objectives in the rehabilitation treatment of stroke.

Acupuncture, a cornerstone of traditional Chinese medicine, is celebrated for its antiquity and widespread use. Recognizing its affordability and minimal side effects, the World Health Organization (WHO) endorses acupuncture as a vital complementary and alternative approach for stroke treatment and care enhancement (9, 10). The “elongated needle,” crafted from flexible stainless steel wire, features a diameter of 0.22–0.40 mm and a length of 70–200 mm (11). Its pine-shaped tip facilitates easy insertion, preventing the formation of barbs or burrs. With its long needle body, strong penetration, and significant stimulation, the elongated needle is well-suited for treating deep-seated blockages and pain. The defining technique of elongated needle is known as piercing needling, which involves adjusting the angle, direction, and depth of needle insertion to hit multiple acupuncture points with a single needle. This practice intensifies the acupuncture effects, thereby showing enhanced rehabilitation outcomes for hemiplegia (12). Compared to conventional techniques, elongated needle acupuncture penetrates more deeply, evokes stronger sensations, and provides more substantial stimulation, effectively initiating muscle contractions. Doing so elicits an antagonistic response, which strengthens muscles in the affected limbs and balances tensions between antagonistic and agonistic muscles, leading to equilibrium. Multiple studies (13–17) indicate that elongated needles substantially improve motor and neurological function as well as balance in patients with post-stroke sequelae. Furthermore, recent research (17–20) suggests that these needles heighten motor neuron excitability in neural reflex arcs through unique forms of peripheral sensory input, boosting muscle strength and tone, diminishing local compression, inhibiting inflammatory factors, and enhancing blood circulation. This contributes significantly to movement improvement, balance regulation, and pain relief.

While many clinical studies have validated the positive effects of elongated needles on post-stroke complications, there is a scarcity of high-quality systematic reviews globally. To date, no evidence robustly supports the safety and efficacy of elongated needle for motor and balance dysfunctions following stroke. This study collates and critically appraises pertinent clinical research literature from Chinese and English sources, using quality assessment and meta-analysis to systematically ascertain the safety and efficacy of elongated needles for treating motor and balance dysfunctions in stroke patients.

## 2 Methods and analysis

### 2.1 Study registration

This systematic review protocol, registered in PROSPERO (No. CRD42024542151), was developed following the Preferred Reporting Items for Systematic Reviews and Meta-Analyses (PRISMA) guidelines.

### 2.2 Inclusion criteria

We used the PICOS framework to formulate the inclusion criteria as follows:

(1) Population: Subjects include patients over 18 years of age diagnosed with stroke-related balance or motor function disorders. The study imposes no restrictions on race, gender, or education level. Diagnosis must adhere to WHO standards (1). (2) Interventions: Use of elongated needles solo or in combination with other therapies (such as conventional acupuncture or rehabilitation therapy). When combined therapies are used, consistency between the experimental and control groups must be maintained. The choice of acupuncture points, duration of needle retention, frequency of acupuncture, and rehabilitation training methods are not restricted. (3) Comparison: Comparisons are made with non-elongated needle treatments including conventional acupuncture, rehabilitation training, pharmacological treatments, or absence of treatment. (4) Primary Outcome Measures: Evaluation of balance and motor abilities. Balance function is assessed using the Berg Balance Scale (BBS), and motor function is evaluated through the Fugl–Meyer Assessment of Motor Function (FMA), which includes upper limb motor scores (FMA-U) and lower limb motor scores (FMA-L). The total potential score is 100 points, with 66 for the upper limbs and 34 for the lower limbs; higher scores indicate better motor function. (5) Secondary Outcome Measures: Assessment includes activities of daily living (ADL) and incidence of adverse events. ADL is evaluated using the Barthel Index (BI) and the Modified Barthel Index (MBI). (6)Studies: Our systematic review and meta-analysis encompass randomized controlled trials (RCTs) that assess the efficacy of elongated needles on balance and motor function. These trials must be published in either English or Chinese.

### 2.3 Exclusion criteria

The following types of articles were excluded: (1) Only the most comprehensive version of duplicate publications was retained. (2) Clinical case reports, review articles, animal studies, conference papers, and papers without control groups. (3) Studies where outcome indicators did not meet the requirements. (4) Studies with unreasonable design aspects such as intervention measures, control measures, or randomization methods. (5) Studies with incomplete data or those for which the full text was unavailable.

### 2.4 Search strategy

The search terms include: Stroke, Post-stroke, Apoplexy, Cerebrovascular Disorder, Brain Ischemia, Intracranial Arterial Disease, Intracranial Hemorrhages; Elongated Needle, Awn Needle, Sharp Long Needle, Mang Zhen, Penetrating Acupuncture, Point-to-Point Acupuncture, Point-Toward-Point Needle Insertion; Randomized Controlled Trial, Controlled Clinical Trial, Randomized, Randomly, etc. Refer to Table 1 for specific search methodologies utilized in PubMed.

**Table 1:** PubMed search strategy.

**Search**	**Query**
#1	“Stroke”[Title/Abstract] OR “Post-stroke”[Title/Abstract] OR “Apoplexy”[Title/Abstract] OR “cerebrovascular disorder”[Title/Abstract] OR “brain ischemia”[Title/Abstract] OR “intracranial arterial disease”[Title/Abstract] OR “intracranial hemorrhages”[Title/Abstract]
#2	“elongated needle”[Title/Abstract] OR “awn needle”[Title/Abstract] OR ((“sharp”[All Fields] OR “sharps”[All Fields]) AND “long needle”[Title/Abstract]) OR (“Mang”[All Fields] AND “zhen”[Title/Abstract]) OR “penetrating acupuncture”[Title/Abstract] OR (“point-to-point”[All Fields] AND “acupuncture”[Title/Abstract]) OR (“Point-toward-point”[All Fields] AND “needle insertion”[Title/Abstract])
#3	“Rehabilitation”[MeSH Terms] OR “pelvic floor rehabilitation”[Title/Abstract] OR “pelvic floor muscle training”[Title/Abstract] OR “PFMT”[Title/Abstract] OR “Kegel movement” [Title/Abstract] OR “Biofeedback training”[Title/Abstract] OR “low-frequency bioelectrical stimulation”[Title/Abstract] OR “vaginal dumbbell”[Title/Abstract]
#4	#1 AND #2 AND #3

### 2.5 Studies' selection and data extraction

Researchers utilized NoteExpress reference management software to eliminate duplicate documents, which was then followed by two researchers independently screening the documents to ascertain their alignment with the inclusion criteria. In the event of any discrepancies, they engaged in a discussion to resolve them; if disagreements persisted, a third researcher would be consulted. Subsequently, the two researchers independently extracted and recorded pertinent information from the full texts of the selected studies, including: (1) Study information: first author, publication year, country, sample size, and risk of bias factors (e.g., randomization methods and blinding); (2) Participant characteristics: age, gender, type of stroke, and disease progression; (3) Experimental group details: acupuncture methods (selection of acupoints, frequency, treatment duration, etc.) and/or additional combined interventions (types, frequency, duration, etc.); (4) Control group details: comparison protocol and/or other combined interventions (types, frequency, duration, etc.); (5) Outcome measures: primary and secondary outcomes. Data were only gathered from the trial arm relevant to the intervention under study if the randomized controlled trial included multiple groups.

### 2.6 Study quality assessment

The methodological quality of all included studies was assessed using the Cochrane Risk of Bias Tool. The studies were evaluated across seven domains to classify bias risk as “high,” “low,” or “unclear”: random sequence generation, allocation concealment, blinding of participants and outcome assessors, selective reporting, incomplete outcome data, and other sources of bias. Each study was reviewed by at least two reviewers. In cases of disagreement, a third independent auditor was consulted for resolution.

### 2.7 GRADE assessment

To assess the methodological quality of our findings, we employed the Graded Recommendations Assessment, Development, and Evaluation (GRADE) framework, which systematically evaluates the certainty of evidence for each outcome (21). The GRADE approach involves a structured assessment of eight key determinants: five factors that may decrease the evidence quality (risk of bias, heterogeneity, indirect evidence, imprecision, and publication bias) and three factors that may increase it (magnitude of effect, dose-response gradient, and plausible confounding). Based on this comprehensive evaluation, the certainty of evidence for individual outcomes was classified into four categories: high, moderate, low, or very low. Details of the GRADE evidence quality assessment are presented in Table 2.

**Table 2:** GRADE of evidence of outcomes of the included trials.

**Quality assessment**							**No of patients**		**Effect**		**Quality**	**Importance**
**No of studies**	**Design**	**Risk of bias**	**Inconsistency**	**Indirectness**	**Imprecision**	**Other considerations**	**Experience**	**Control**	**Relative (95% CI)**	**Absolute**		
**BBS (Better indicated by lower values)**												
10	Randomized trials	No serious risk of bias	No serious inconsistency	No serious indirectness	Serious^1^	None	371	369	-	MD 6.34 higher (4.8 to 7.89 higher)	⊕⊕⊕○ MODERATE	IMPORTANT
**FMA (Better indicated by lower values)**												
4	Randomized trials	No serious risk of bias	Serious^2^	No serious indirectness	Very serious^3^	None	145	145	-	MD 10.23 higher (3.57 to 16.89 higher)	⊕○○○ VERY LOW	IMPORTANT
**FMA-U (Better indicated by lower values)**												
6	Randomized trials	No serious risk of bias	Serious^2^	No serious indirectness	Serious^1^	None	196	198	-	MD 4.42 higher (2.21 to 6.63 higher)	⊕⊕○○ LOW	IMPORTANT
**FMA-L (Better indicated by lower values)**												
1	Randomized trials	Serious^4^	Serious^2^	No serious indirectness	No serious imprecision	None	42	42	-	MD 3.11 higher (2.56 to 3.66 higher)	⊕⊕○○ LOW	IMPORTANT
**MBI (Better indicated by lower values)**												
4	Randomized trials	No serious risk of bias	Serious^2^	No serious indirectness	Serious^1^	None	126	124	-	MD 8.35 higher (5.8 to 10.91 higher)	⊕⊕○○ LOW	IMPORTANT
**BI (Better indicated by lower values)**												
6	Randomized trials	No serious risk of bias	Serious^2^	No serious indirectness	No serious imprecision	None	197	197	-	MD 6.21 higher (5.34 to 7.08 higher)	⊕⊕⊕○ MODERATE	IMPORTANT

**GRADE Working Group grades of evidence**

**High quality:** Further research is very unlikely to change our confidence in the estimate of effect.

**Moderate quality:** Further research is likely to have an important impact on our confidence in the estimate of effect and may change the estimate.

**Low quality:** Further research is very likely to have an important impact on our confidence in the estimate of effect and is likely to change the estimate.

**Very low quality:** We are very uncertain about the estimate.

**1:** The included studies showed moderate heterogeneity (50% < *I*^2^ < 75%) in the heterogeneity test.

**2:** The total sample size of the included studies was insufficient, resulting in wide confidence intervals.

**3:** The included studies exhibited considerable heterogeneity (*I*^2^ ≥ 75%) in the heterogeneity test.

**4:** Only one small-sample study was included in the analysis.

### 2.8 Data analysis

A quantitative analysis of the data from the included studies was conducted using RevMan v5.4 and Stata 16.0 software. 95% Confidence Intervals (95% CI) were employed as statistical measures. Mean Difference (MD) was utilized for analyzing continuous outcomes measured in identical units; otherwise, Standardized Mean Difference (SMD) was applied. The *I*^2^ test was used to assess heterogeneity. Heterogeneity was considered significant when *I*^2^ > 50% and *p* < 0.1, prompting either sensitivity analysis or subgroup analysis. A comparison of combined effect sizes revealed a significant difference between the observation and control groups (*P* < 0.05). When the 95% CI of the combined effect size encompasses 1 or 0, it suggests no statistically significant difference between the groups; conversely, exclusion of 1 or 0 from the 95% CI indicates a statistically significant difference. For outcome indicators with *n* ≥ 10 studies, the Begg test and funnel plot were utilized to assess publication bias.

## 3 Results

### 3.1 Selection process

**Figure 1:**
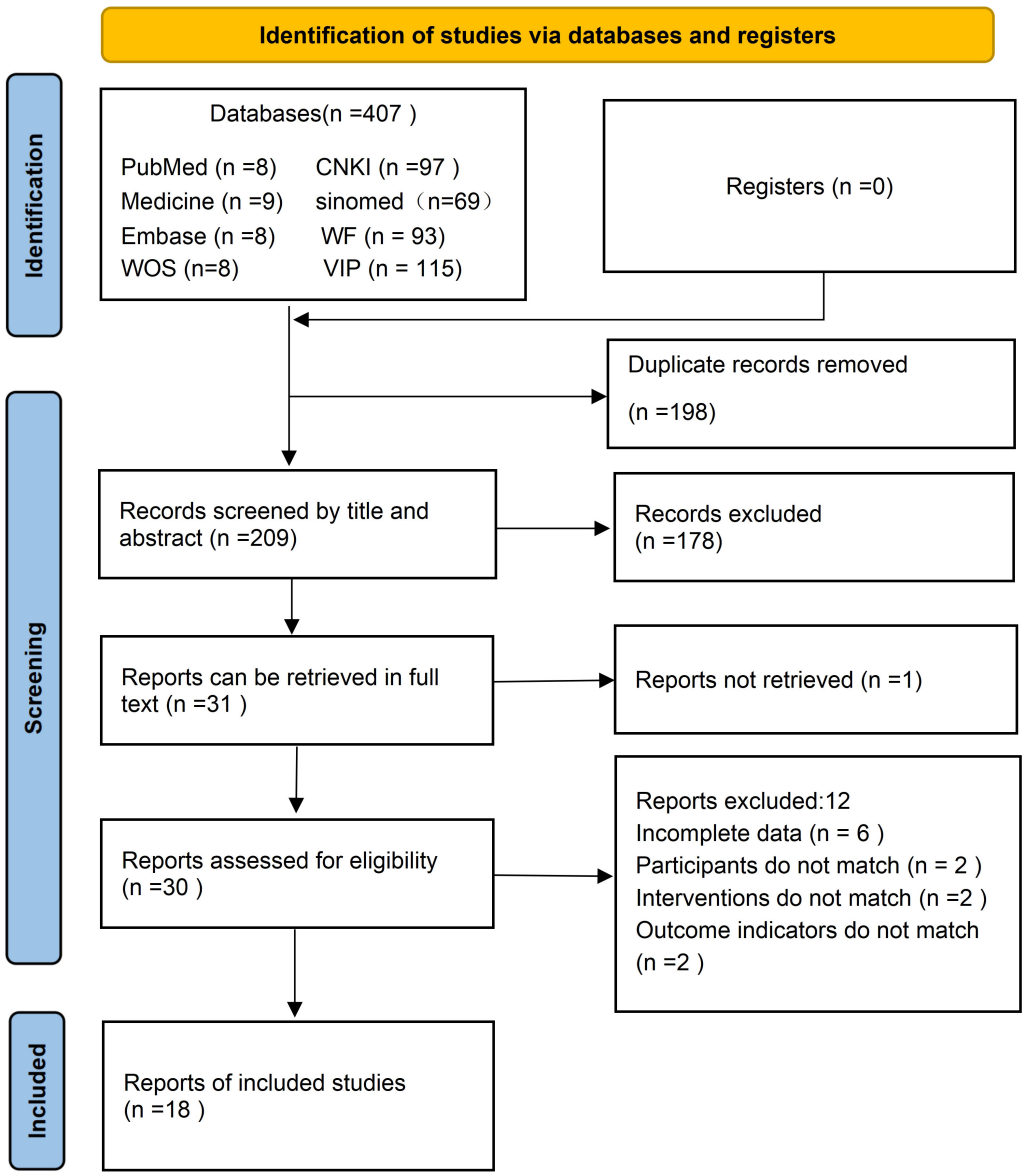
Literature screening flowchart.

Figure 1 illustrates the literature selection process. Initially, 407 articles from various Chinese and English databases were identified. After eliminating 199 duplicates, 178 articles were excluded based on their titles and abstracts. Furthermore, one article was excluded due to the unavailability of the full text. Following a full-text review of the remaining 30 articles, 18 met the eligibility criteria and were included in the final analysis. All selected articles were in Chinese, and no relevant studies on elongated needle for balance and motor function disorders in stroke patients were identified in international databases.

### 3.2 Basic characteristics of the included studies

A total of 18 studies were included (15–18, 22–35), involving 1,230 participants, with 615 in the experimental group and 615 in the control group. The baseline data for the experimental and control groups in the included studies were essentially consistent and comparable. The basic characteristics of the included studies are presented in Table 2. Among these, five studies (18, 25, 27, 34, 35) utilized conventional acupuncture as an intervention for the control group. Four studies (15, 17, 29, 32) implemented rehabilitation training, while another four studies (16, 22, 24, 28) used a combination of conventional acupuncture and rehabilitation training. Two studies (23, 33) employed oral traditional Chinese medicine, and three studies (26, 30, 31) applied alternative therapies (electroacupuncture, bloodletting cupping, acupoint application) in the control group. Table 3 details the characteristics of the included studies.

**Table 3:** Literature data feature extraction table.

**Reference**	**Sample size (E/C)**	**Age (E/C)**	**Gender (M/F)**	**Disease duration**	**Intervention**			**Outcomes**
					**Methods**	**Frequency**	**Duration**	
Mao (18)	60 (30/30)	E: 58.20 ± 9.60 C: 60.03 ± 9.68	E: 19/11 C: 21/9	E:89.47 ± 51.45 C:87.77 ± 56.52 (day)	E: EN C: CA	E: 40 min/d, 6 d/w C: 40 min/d, 6 d/w	4 w	;
He (32)	60 (30/30)	E: < 40(1);40–69(26);>70(3) C: < 40(1);40–69(24); >70(5)	E: 19/11 C: 20/10	NA	E: EN+CA; RT; C: CA; RT	NA	20 d	;
Tian (34)	60 (30/30)	E: 57.4 ± 7.2 C: 58.0 ± 8.9	E: 15/15 C: 14/16	E: 55.1 5.15.4 C: 46.7 6.18.5 (day)	E: EN; +CA T:CA	E: 30 min/d, 6 d/w C: 30 min/d, 6 d/w	4 w	;
Yuan (24)	70 (35/35)	E: 56.34 ± 6.76 C: 54.51 ± 6.93	E: 17/18 C: 20/15	E:5.41 .41.89 C:5.99 .92.23 (month)	E: EN C: RT	E: 40 min/d, 6 d/w C: 45 min/d, 6 d/w	4 w	;
Gao (27)	60 (30/30)	E: 57.40 ± 7.29 C: 56.40 ± 7.47	E: 19/11 C: 18/12	E: 58.37 8.13.57 C: 57.70 7.11.18 (day)	E: EN+CA; C: CA;	E: 30 min/d, 6 d/w C: 30 min/d, 6 d/w	6 w	;
Jiang and Cao (25)	60 (30/30)	E: 56.80 ± 5.64 C: 58.40 ± 7.57	E: 19/11 C: 23/7	E: 5.95 .92.50 C: 4.57 .53.47 (month)	E: EN; C: CA;	E: 40 min/d, 4 d/w C: 40 min/d, 4 d/w	40 d	;
He and Zhou (35)	60 (30/30)	E: 52.00 ± 11.18 C: 50.47 ± 12.04	E: 14/16 C: 16/14	E: 59.13 9.28.79 C: 56.80 6.21.31 (day)	E: EN+CA C: CA;	E: 30 min/d, 6 d/w C: 30 min/d, 6 d/w	4 w	
Zhao et al. (17)	60 (30/30)	E: 62.0 ± 5.0 C: 59.0 ± 9.0	E: 21/9 C: 19/11	E: 105.7 0538.7 C: 107.0 0730.2 (day)	E: EN+RT C: CA+RT	E: 30 min/d, 5 d/w; 40 min/d, 5 d/w C: 30 min/d, 5 d/w; 40 min/d, 5 d/w	4 w	
Zhou et al. (26)	56 (29/27)	E: 58.76 ± 10.11 C: 58.52 ± 10.83	E: 14/15 C: 12/15	E: 58.76 8.10.11 C: 58.52 8.10.83 (day)	E: EN+EA C: EA	E: 30 min/d, 7 d/w C: 30 min/d, 7 d/w	2w	;
Guo et al. (22)	96(48/48)	E: 61.23 ± 5.49 C: 62.23 ± 4.47	E: 25/23 C: 26/22	E: 38.96 8.7.25 C: 36.74 6.7.78 (day)	E: EN,RT C: RT	E: 2^*^30 min/d, 7 d/w; 2^*^30 min/d, 7 d/w C: 2^*^30 min/d, 7 d/w	4w	
Zhan et al. (23)	84(42/42)	E: 64.75 ± 8.26 C: 64.31 ± 8.68	E: 25/27 C: 23/19	NA	E: EN; CM(HGWD) C: CM(HGWD)	E: 40 min/d, 5 d/w, 2^*^100ml/d C: 2^*^100ml/d	4w	
Zeng et al. (33)	110(55/55)	E: 57.24 ± 4.65 C: 58.01 ± 4.53	E: 30/25 C: 31/24	NA	E: EN; CM(WTD) C: CM(WTD)	E: 30 min/d, 4 d/w, 2^*^150ml/d C: 30 min/d, 4 d/w,2^*^150ml/d	8w	;
Pan (29)	60 (30/30)	E: 56.70 ± 9.00 C: 58.47 ± 8.35	E: 17/13 C: 18/12	E: 73.56 3.30.22 C: 81.80 1.33.69 (day)	E: EN+RT C: CA+RT	E: 30 min/d, 4 d/w; 40 min/d, 4 d/w C: 30 min/d, 4 d/w; 40 min/d, 4 d/w	4w	
Zhi (30)	60 (30/30)	E: 56.70 ± 9.00 C: 58.47 ± 8.35	E: 17/13 C: 16/14	E: 73.56 3.30.22 C: 81.80 1.33.69 (day)	E: EN; PAC C: CA; PAC	E: 30 min/d, 6 d/w; 4 d/w C: 30 min/d, 6 d/w; 4 d/w	6w	
Chen et al. (16)	84(42/42)	E: 59.00 ± 3.00 C: 58.00 ± 3.00	E: 25/17 C: 28/14	NA	E: EN; RT C: RT	E: 30 min/d, 6 d/w; 20-35 min/d C: 20-35 min/d	4w	; ;
Pu et al. (31)	60 (30/30)	E: 58.42 ± 4.98 C: 59.15 ± 4.42	E: 17/13 C: 18/12	E: 41.08 1.5.41 C: 40.89 0.4.62 ()	E: EN; AA C: AA	E: 30 min/d, 6 d/w; 7 d/w C: 7 d/w	4w	;
Zhang et al. (15)	74 (37/37)	E: 59.73 ± 5.54 C: 59.41 ± 6.08	E: 24/13 C: 25/12	E: 28.90 8.12.30 C: 29.40 9.13.30 (day)	E: EN+CA; RT C: CA; RT	E: 30 min/d, 7 d/w; 30 min/d, 7 d/w C: 30 min/d, 7 d/w; 30 min/d, 7 d/w;	4w	;
Liu et al. (28)	56 (27/29)	E: 64.50 ± 5.60 C: 66.80 ± 6.20	NA	NA	E: EN,RT C: RT	E: 30 min/d, 6 d/w; 40 min/d, 6 d/w C: 40 min/d, 6 d/w	4w	

E, experimental group; C, control group; F, male; M, male; d, day; w, week; EN, elongated needle; CA, conventional acupuncture; RT, Rehabilitation training; EA, electroacupuncture; CM: Chinese medicine; HGWD, Huangqi Guizhi Wuwu Decoction; WTD, Wenjing Tongmai Decoction; PAC: Pricking and cupping; AA, acupoint application; FMA; FMA-u; FMA-L; BBS; BI; MBI.

### 3.3 Study quality assessment (risk of bias)

**Figure 2:**
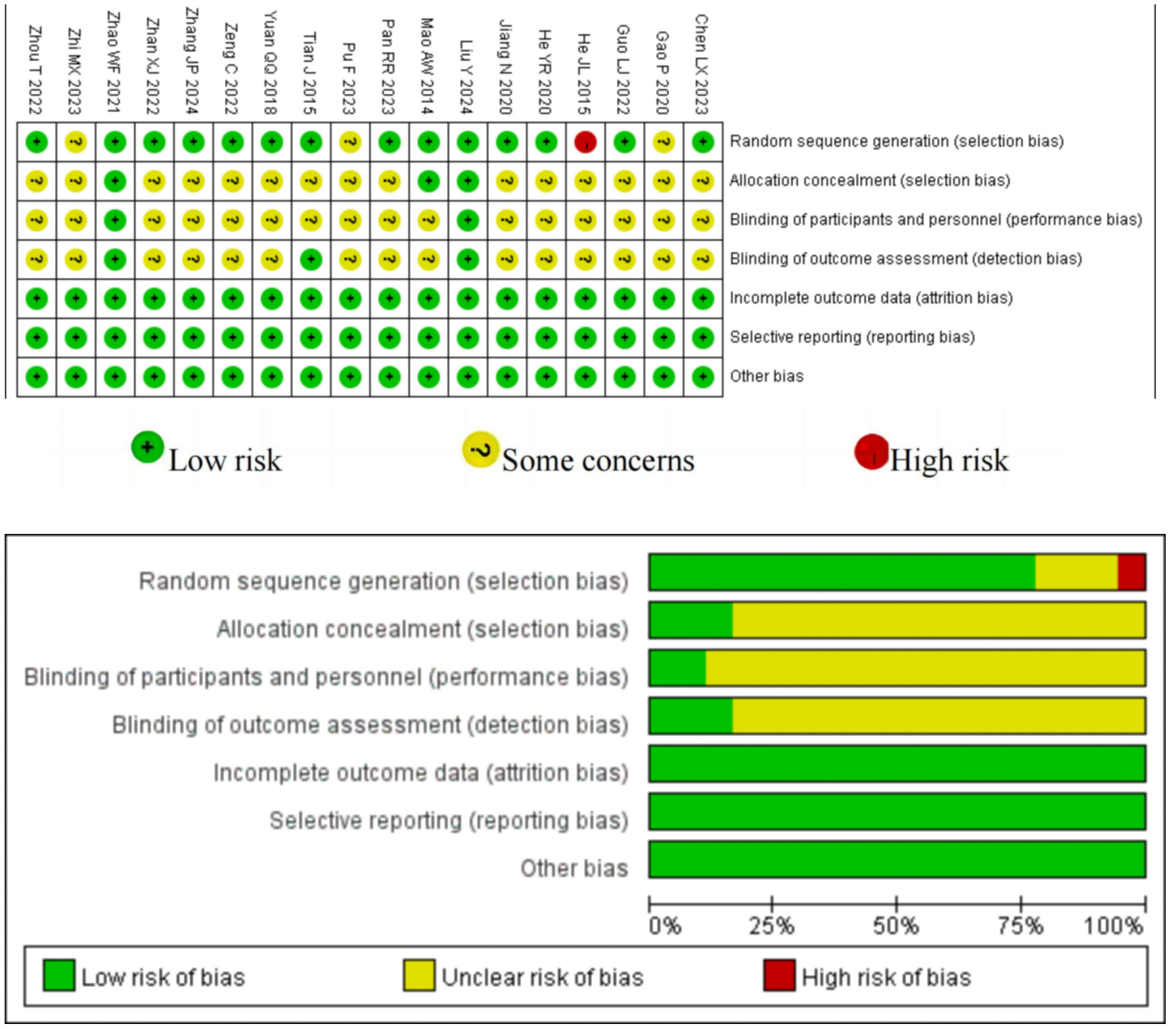
Risk of bias assessment of included studies.

All studies employed the Cochrane Handbook for Systematic Reviews of Interventions to evaluate bias risk. Each incorporated study utilized randomization; 14 provided explicit details on the random sequence generation process, three did not specify the randomization method, and one was considered high risk due to improper randomization. Two studies mentioned adequate allocation concealment and were deemed low risk, whereas the other 16 studies failed to mention adequate allocation. Given the unique nature of acupuncture interventions, blinding participants and assessors poses a significant challenge. Nonetheless, two studies managed to blind participants to reduce bias risk, three outlined specific methods for blinding assessors, and the remaining studies lacked details on blinding procedures. All included RCTs preserved data integrity without evidence of selective outcome reporting. The results of the quality assessment are depicted in Figure 2.

### 3.4 Meta-analysis

#### 3.4.1 Primary outcome measures

##### 3.4.1.1 Balance ability assessment (BBS)

**Figure 3:**
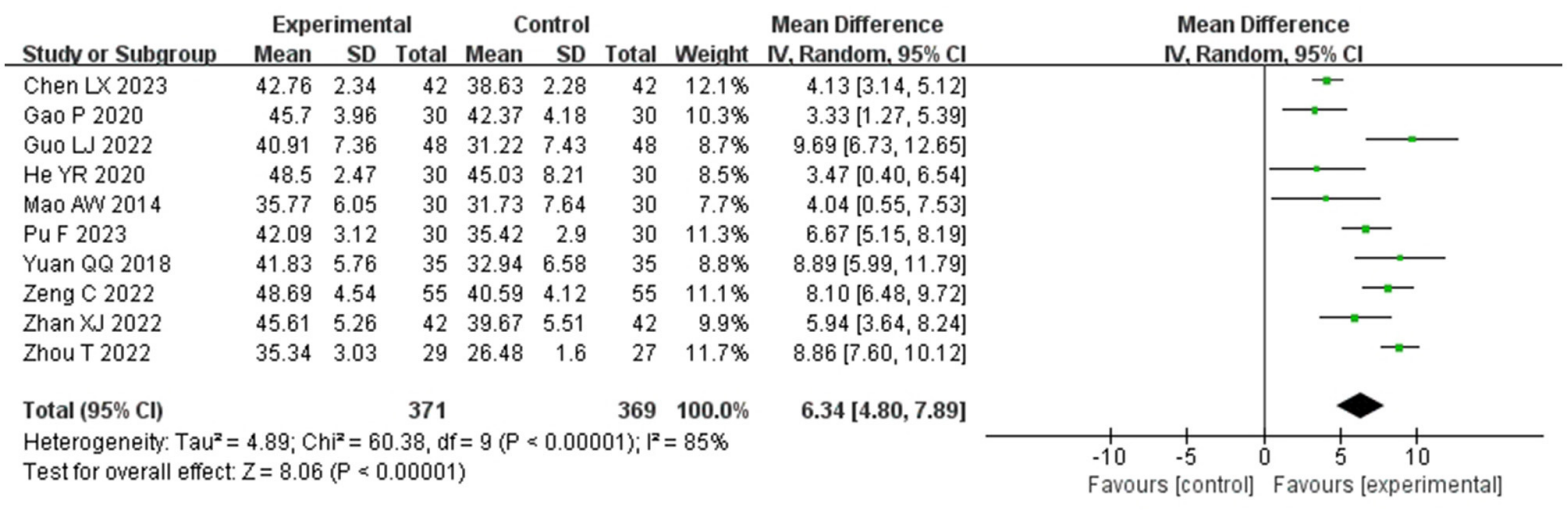
Forest plot of BBS comparisons between groups.

Figure 3 presents the meta-analysis of BBS scores comparing the experimental group and the control group. Included in this analysis were 10 studies (16, 18, 22–24, 26, 27, 31, 33, 35), involving 740 patients. Due to high statistical heterogeneity among the studies (P > 75%), a random effects model was applied. The findings revealed a significant difference between the experimental and control groups (MD = 6.34, *P* < 0.001, *I*^2^ = 85, 95% CI = 4.80–7.89), indicating that the experimental group was more effective in enhancing balance function. To assess the robustness of these results and the impact of each study on the overall effect size, a sensitivity analysis was performed by sequentially removing one study at a time and recalculating the results; this confirmed the stability of the meta-analysis findings.

**Figure 4:**
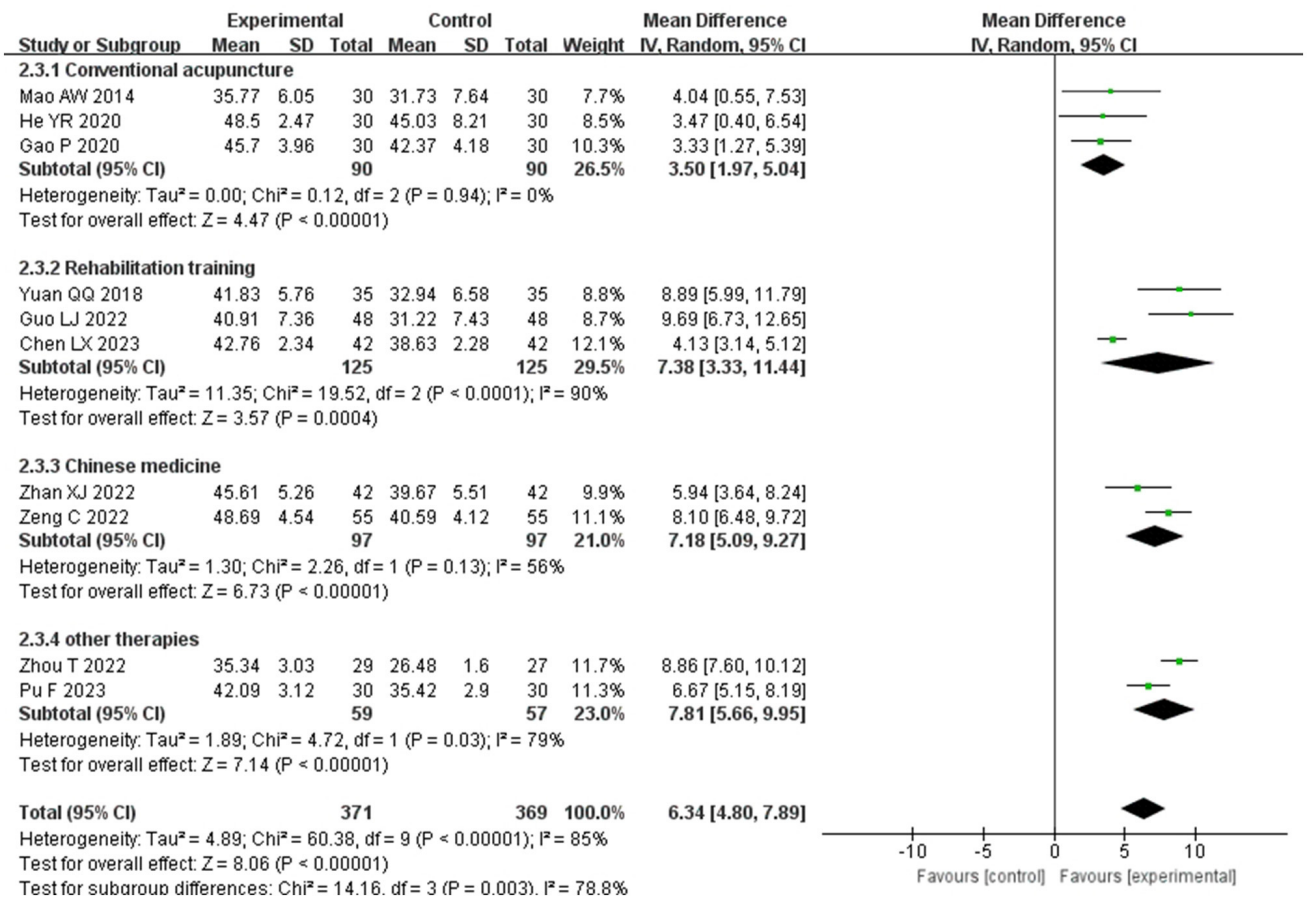
Forest plot of BBS subgroup analyses stratified by control types.

A subgroup analysis was conducted, dividing the control group into four categories: conventional acupuncture, rehabilitation training, Chinese medicine, and other therapies, as depicted in Figure 4. The experimental group demonstrated superior Berg Balance Scale (BBS) scores compared to the control groups: conventional acupuncture (MD = 3.50, *P* < 0.00001, *I*^2^ = 0%, 95%CI = 1.75–5.04), rehabilitation training (MD = 7.38, *p* = 0.0004, *I*^2^ = 90%, 95%CI = 3.33–11.44), Chinese medicine (MD = 7.18, *P* < 0.00001, *I*^2^ = 56%, 95% CI = 5.09–9.27), and other therapies (MD = 7.81, *P* < 0.00001, *I*^2^ = 79%, 95%CI = 5.66–9.95). These results suggest that elongated needle, either alone or in combination with other methods, is more effective in enhancing balance in post-stroke patients than conventional acupuncture, rehabilitation training, or Chinese medicine alone.

##### 3.4.1.2 Motor ability assessment (FMA, FMA-U, FMA-L)

**Figure 5:**
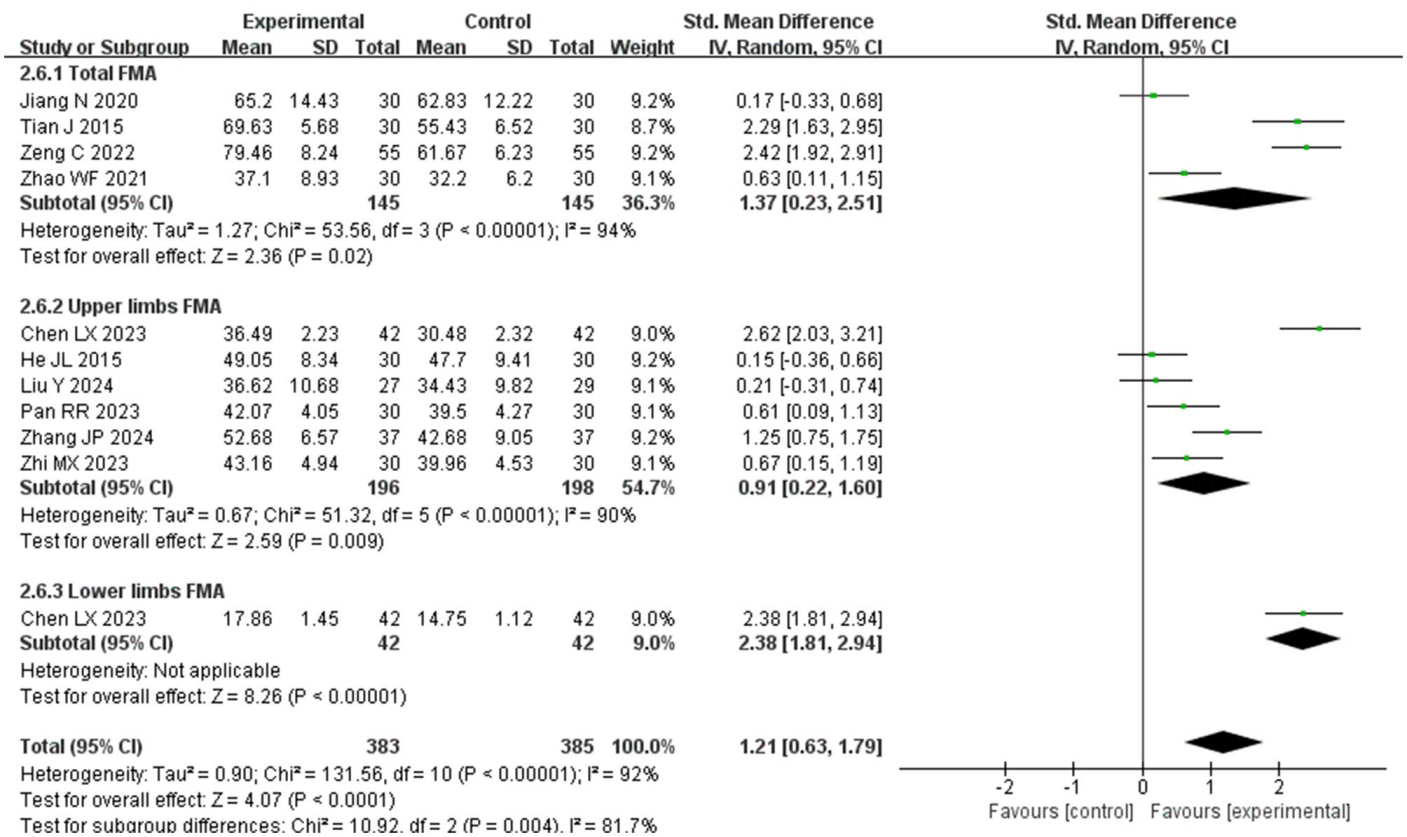
Forest plot of motor ability comparisons between groups.

Figure 5 presents the comprehensive meta-analysis of the Fugl-Meyer Assessment (FMA) scores comparing the experimental and control groups. The analysis included 10 studies (15–17, 25, 28–30, 32–34), encompassing 684 patients. Among these, 4 studies (17, 25, 33, 34) reported total FMA scores, 6 studies (15, 16, 28–30, 32) focused on upper limb FMA scores, and 116 study addressed the lower limb FMA scores. Subgroup analyses were stratified by different sites. Due to significant heterogeneity among the studies, a random effects model was applied. Additionally, scores were standardized using the standard mean difference (SMD) method to facilitate comparison, since the total FMA score is out of 100 points, the upper limb out of 66 points, and the lower limb out of 34 points. The results indicated a more considerable improvement in the experimental group compared to the control group (SMD = 1.21, *P* < 0.00001, *I*^2^ = 92%, 95% CI = 0.63–1.79). Elongated needles alone or in combination with other therapies significantly enhanced the overall FMA score by 1.37 points (95% CI = 0.23–2.51), upper limb scores by 0.91 points (95% CI = 0.22–1.60), and lower limb scores by 2.38 points (95% CI = 1.81–2.94). These findings suggest that elongated needles, alone or in conjunction with other therapies, significantly improves motor function in post-stroke patients, particularly benefiting lower limb motor skills.

#### 3.4.2 Secondary outcome measures

##### 3.4.2.1 Assessment of activities of daily living (BI, MBI)

**Figure 6:**
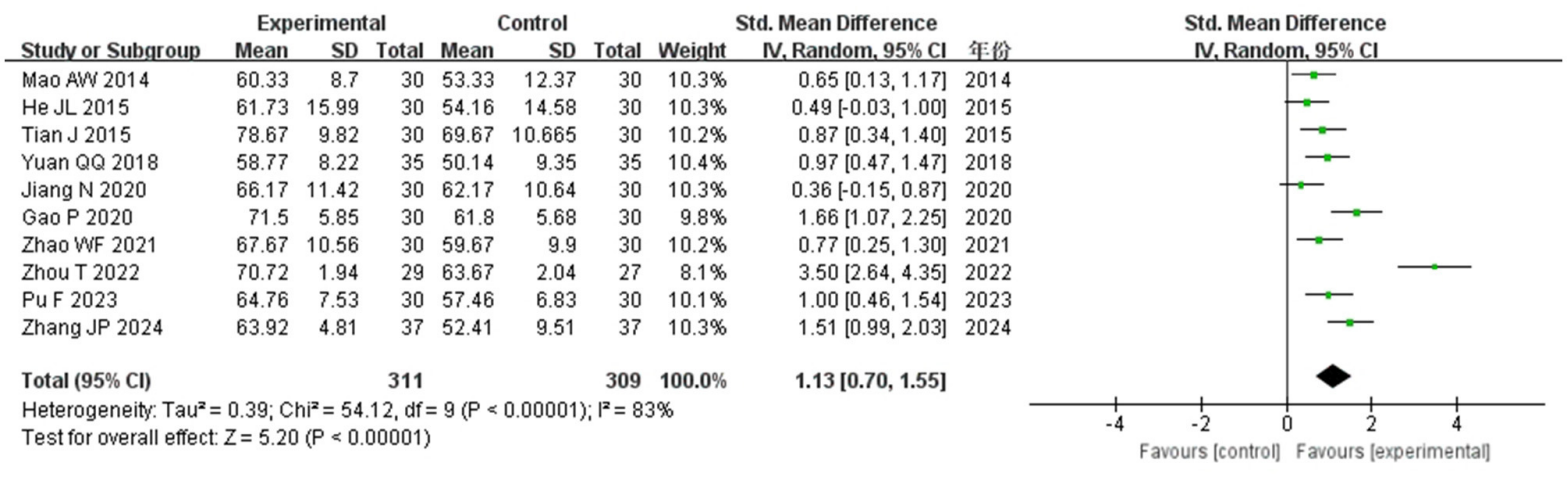
Forest plot of ADL comparisons between groups.

Figure 6 presents a meta-analysis of ADL differences between experimental and control groups, incorporating data from 10 studies (15, 17, 18, 24–27, 31, 32, 34) involving 644 patients. Due to the use of two distinct scales (BI and MBI) in the included RCT studies for ADL scores, the standardized mean difference (SMD) was employed for the analysis. The results revealed a significant disparity between the groups (SMD = 1.13, *I*^2^ > 75%, *P* < 0.001). Sensitivity analysis confirmed the robustness of the effect size (SMD = 1.13), which remained consistent even after exclusions, with the 95% CI for the SMD ranging from 0.73 to 1.55, thus indicating stability in the study outcomes.

**Figure 7:**
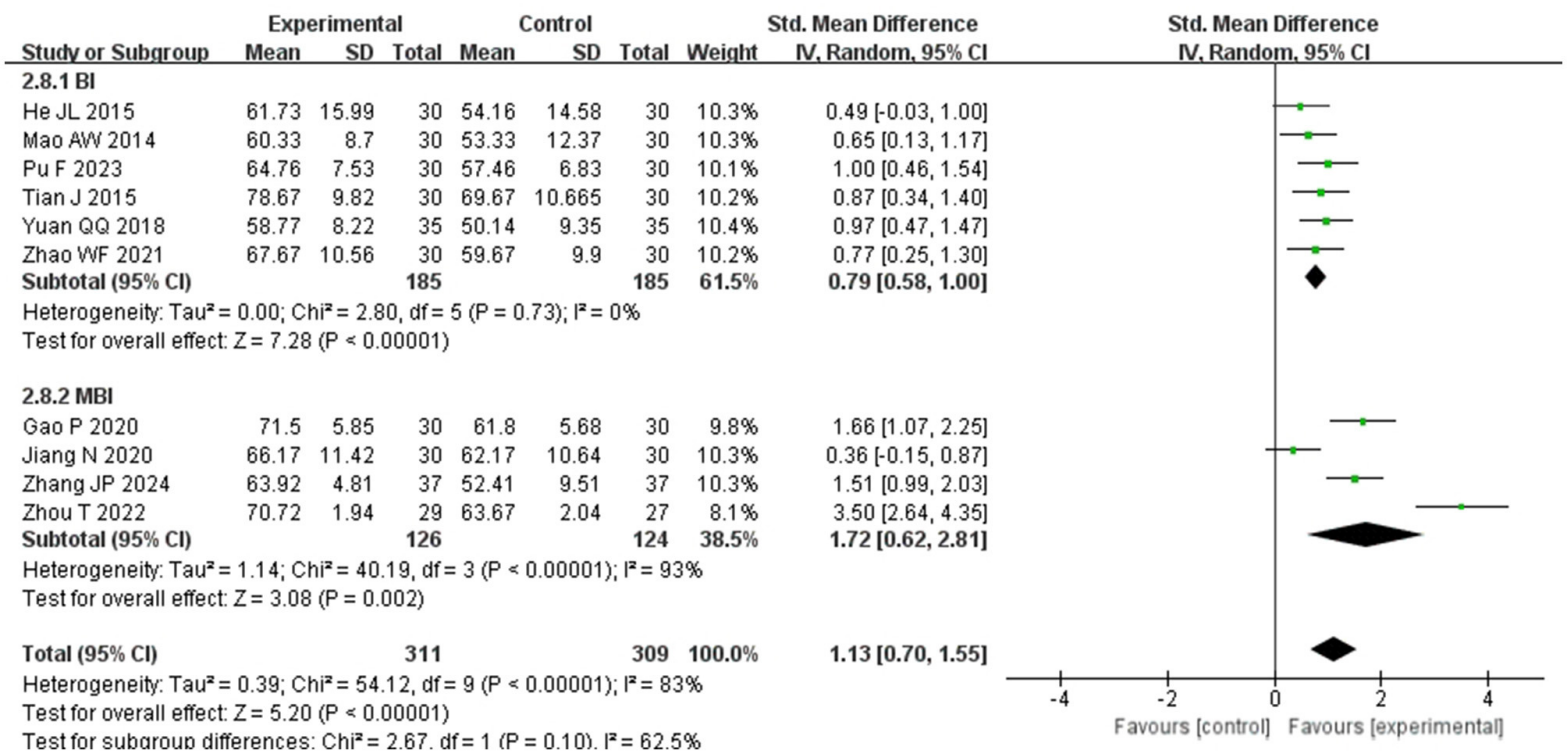
Forest plot of ADL subgroup analyses stratified by assessment scales.

**Figure 8:**
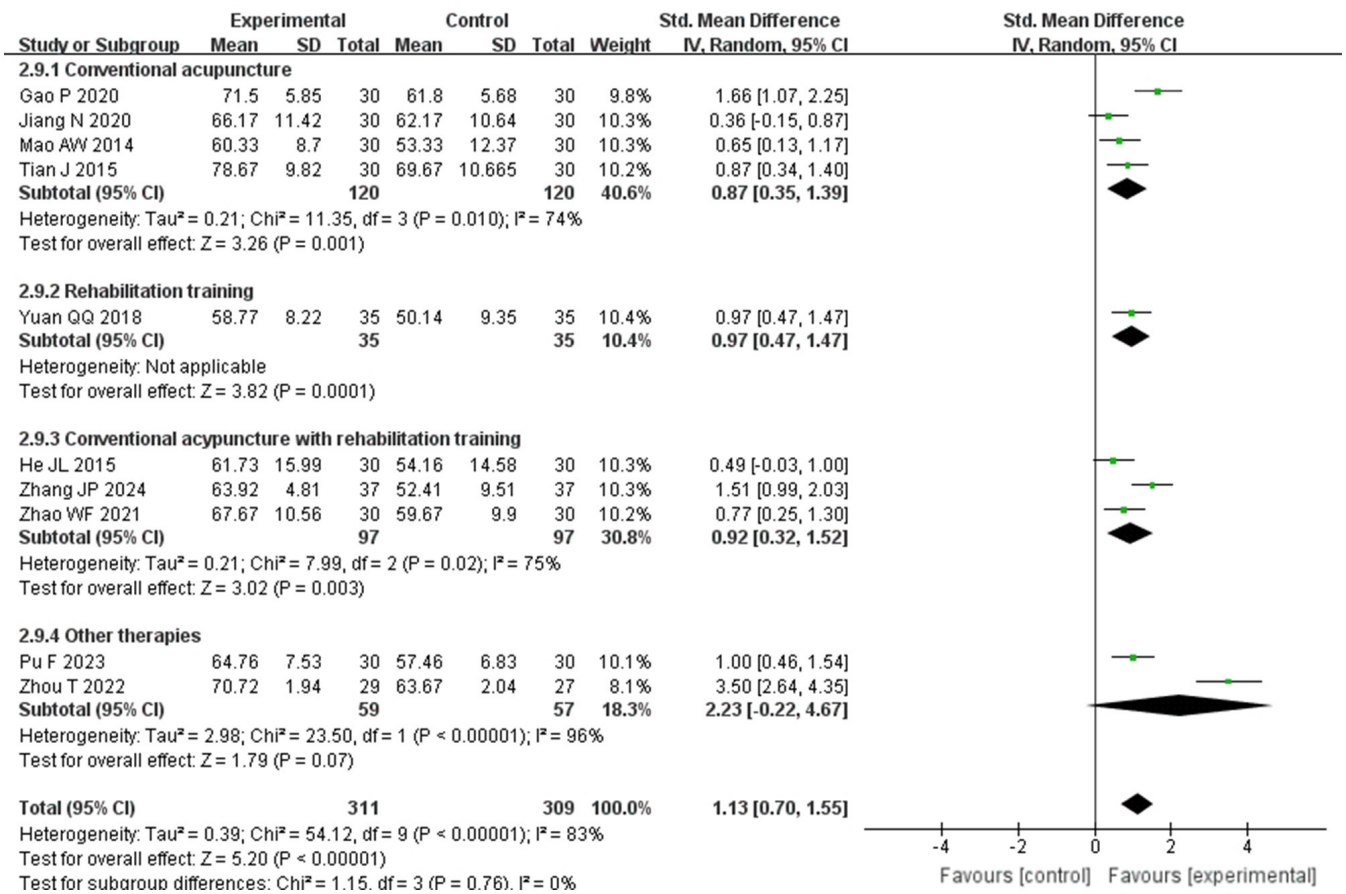
Forest plot of ADL subgroup analyses stratified by control interventions.

Subgroup analysis based on the scale used to assess ADL scores was carried out within the study groups. The analysis revealed that the ADL scores of the experimental group were significantly higher than those of the control group in both the BI group (SMD = 0.79, *P* < 0.00001, *I*^2^ = 0%, 95%CI = 0.58–1.00) and the MBI group (SMD = 1.72, *P* < =0.002, *I*^2^ = 93%, 95%CI = 0.62–2.81), demonstrating reduced heterogeneity within the study, as illustrated in Figure 7. This suggests that the observed high heterogeneity may be attributed to the varying scales utilized for ADL scoring. Furthermore, the control group was categorized into subgroups based on their treatments: conventional acupuncture, rehabilitation training, a combination of both, and other therapies. Comparative analyses showed superior ADL scores in the experimental group over the subgroup receiving conventional acupuncture alone (SMD = 0.87, *p* = 0.001, *I*^2^ = 74%, 95%CI = 0.35–1.39), the rehabilitation training subgroup (SMD = 0.97, *p* = 0.0001, *I*^2^ = 0%, 95%CI = 0.47–1.47), and the combined treatment subgroup (SMD = 0.92, *P* < 0.003, *I*^2^ = 75%, 95%CI = 0.32–1.52). However, there was no statistically significant difference when the experimental group was compared with the subgroup undergoing other therapies (SMD = 2.23, *p* = 0.07, *I*^2^ = 96%, 95%CI = −0.22 to 4.67). These findings indicate that elongated needle, either alone or in combination with other treatments, is more effective than conventional acupuncture, rehabilitation training, or their combination in enhancing daily living abilities among post-stroke patients. Nonetheless, in comparison to other modalities such as electro-acupuncture or acupoint application, more evidence is required to confirm if elongated needle acupuncture is actually superior in improving daily living abilities in post-stroke patients. Refer to Figure 8 for further details.

##### 3.4.2.2 Adverse event

Two studies (15, 31) reported adverse reactions. In one study (31), one participant in the experimental group experienced needle-induced dizziness, while another developed a subcutaneous hematoma. Another study (15) documented a single case of a subcutaneous hematoma in the experimental group. The remaining 16 studies reported no related adverse reactions, suggesting the safety of mang needle therapy.

### 3.5 Publication bias

**Figure 9:**
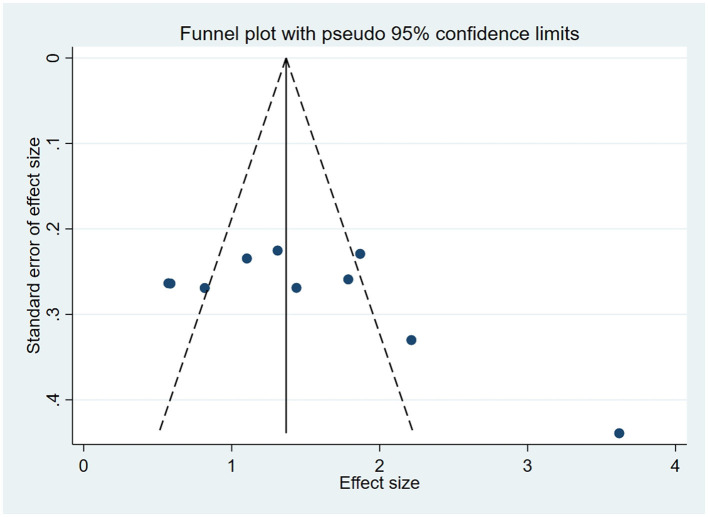
Funnel plot of BBS.

**Figure 10:**
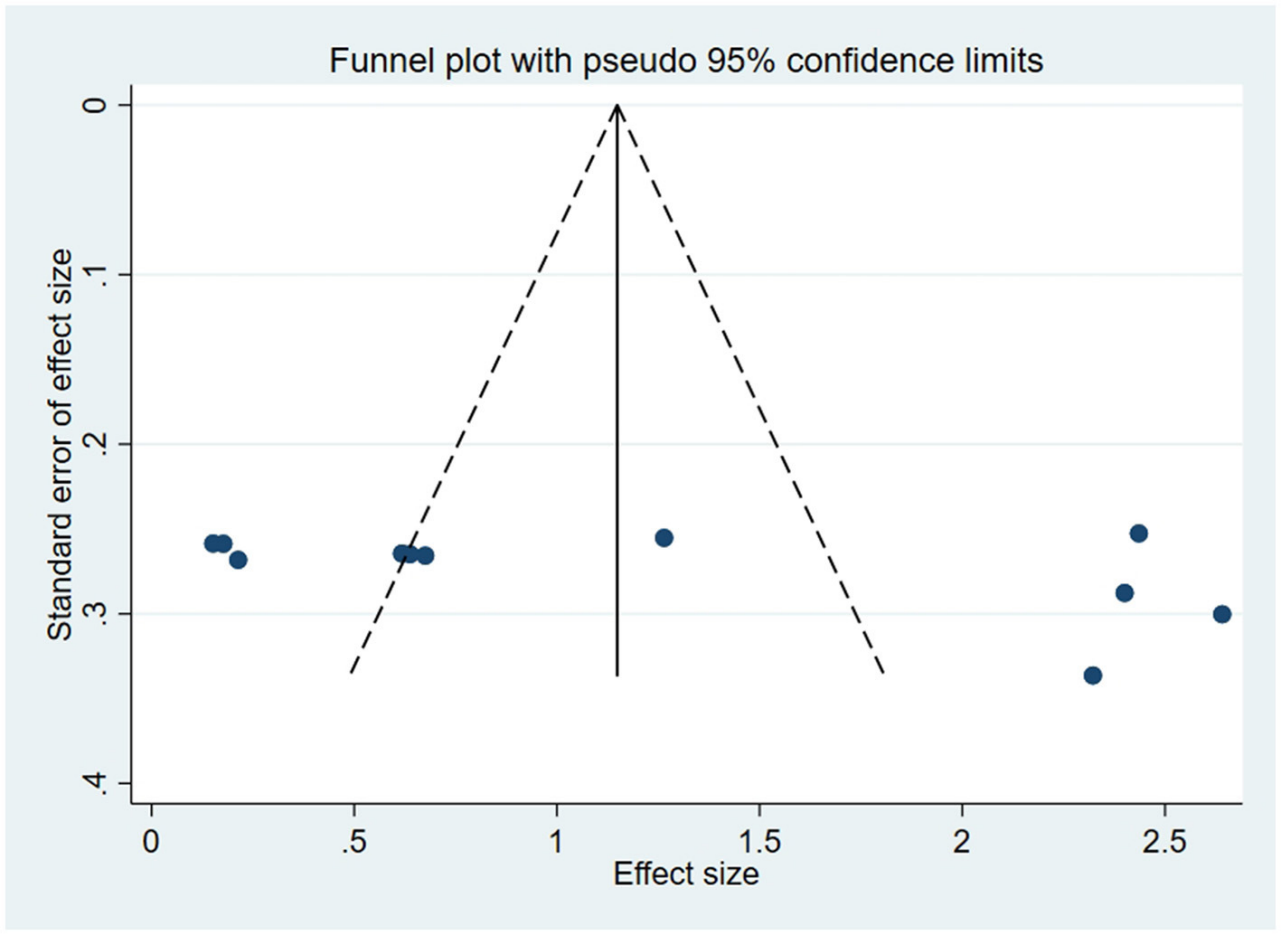
Funnel plot of FMA.

**Figure 11:**
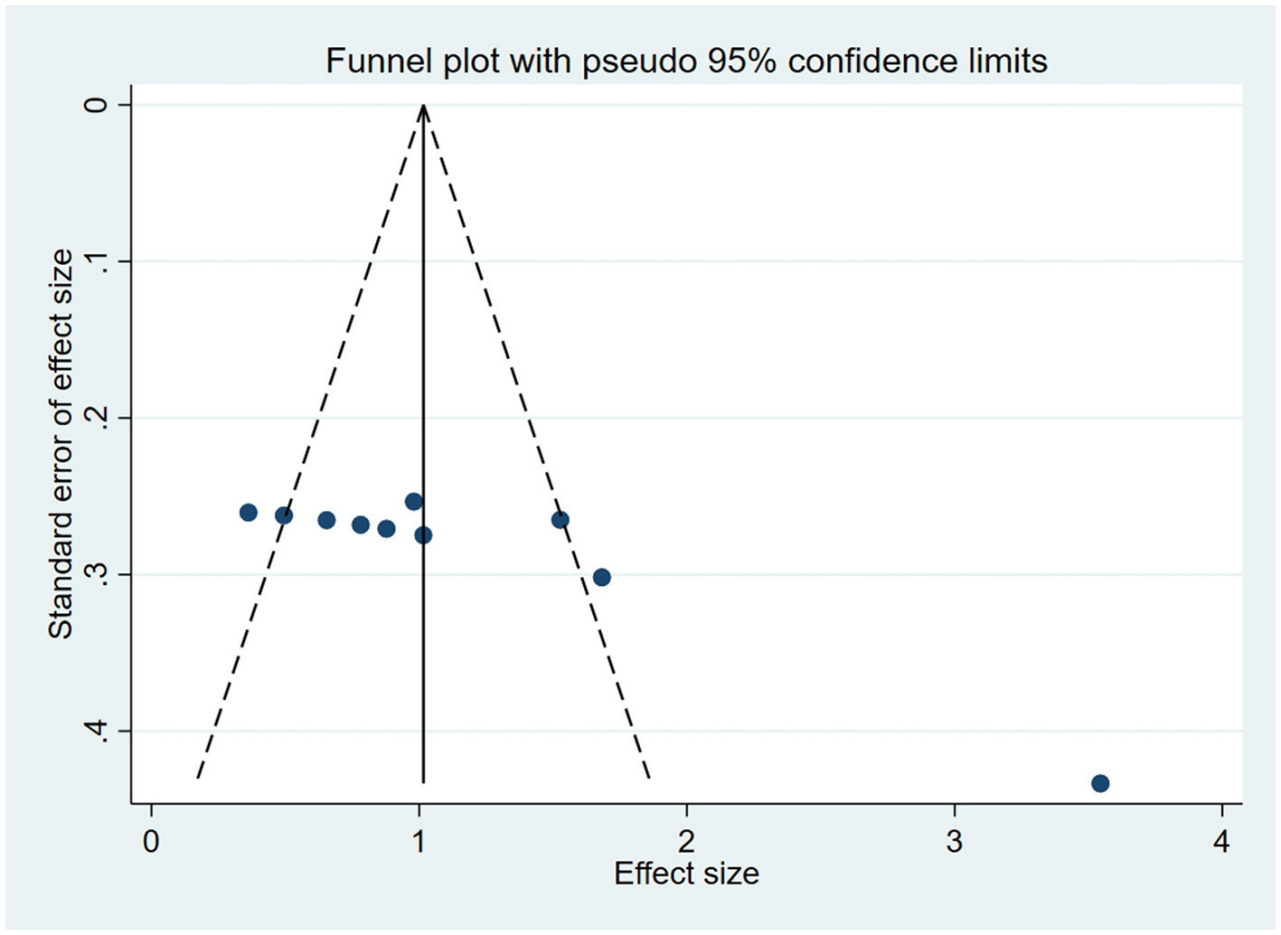
Funnel plot of ADL.

**Figure 12:**
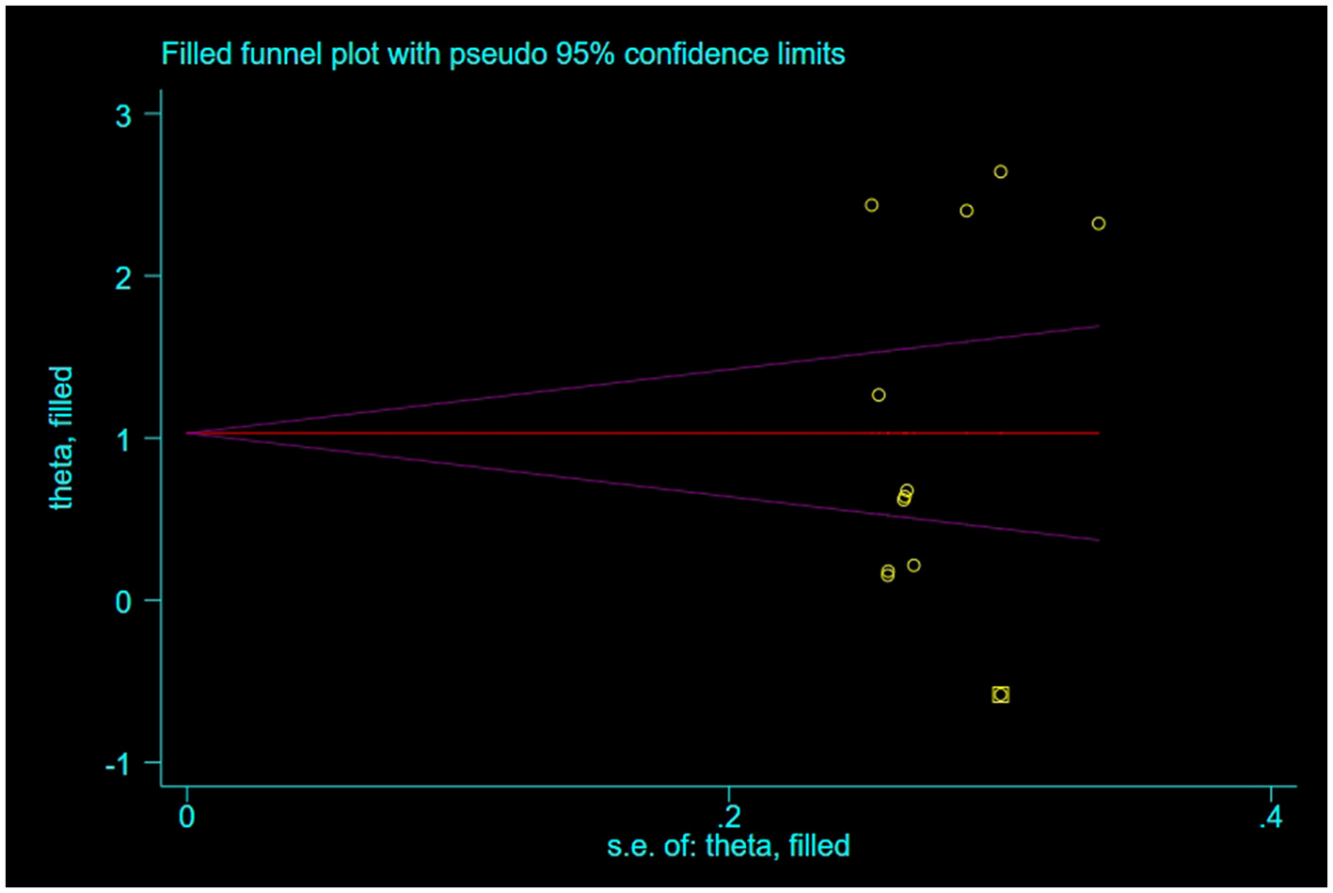
Trim-and-fill adjusted funnel plot of FMA.

**Figure 13:**
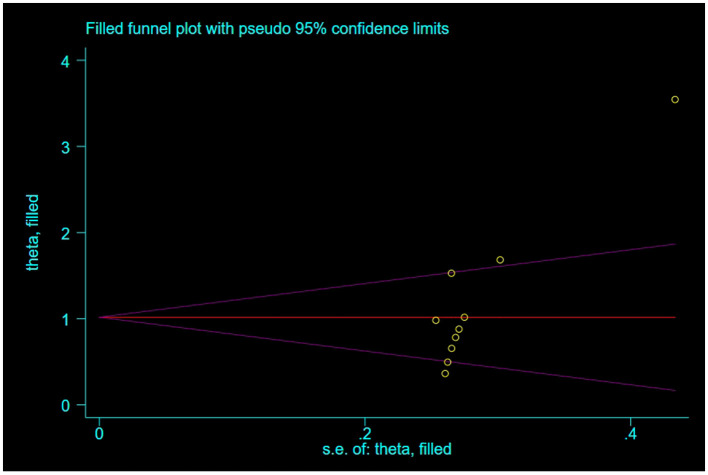
Trim-and-fill adjusted funnel plot of ADL.

For outcome indicators supported by a substantial number of research studies (*n* ≥ 10), publication bias was assessed. Symmetry in funnel plots was analyzed to evaluate publication bias in studies concerning BBS, FMA, and ADL, as depicted in Figures 9–11; symmetric funnel plots suggest an absence of publication bias. Based on the Begg's quantitative test applied to these funnel plots, the result for BBS was *P* = 0.47 (*P* > 0.05), indicating symmetry and suggesting no significant publication bias; the result for FMA was *P* = 0.35 (*P* > 0.05), yet the funnel plot displayed asymmetry, implying potential publication bias; the result for ADL was *P* = 0.02 (*P* < 0.05), with the plot showing notable asymmetry, indicating potential publication bias. To address these asymmetries, the trim-and-fill method was utilized (Figures 10, 11). Post-adjustment, the FMA analysis, depicted in Figure 12, added one study but did not alter the overall results, indicating robust combined outcomes. Similarly, the ADL analysis, shown in Figure 13, underwent no changes after the trim method was applied, confirming the stability of the results. Despite some indications of publication bias, the study's combined results remain robust.

## 4 Discussion

It is estimated that by 2030, over 70 million individuals worldwide will be stroke survivors (36). This projection may impose considerable demands on neurologists and rehabilitation physicians. As a result, the rehabilitation of stroke survivors has garnered significant attention. Acupuncture, a traditional Chinese medicine technique that is non-pharmacological, is increasingly being used to tackle the aftermath of strokes. The elongated needle, a specialized acupuncture tool, typically measures 0.22–0.40 mm in diameter and 70–200 mm in length, making it thinner and longer than the conventional filiform needles, which are usually 25–75 mm long. The technique for using the elongated needle involves inserting it into one acupuncture point and then seamlessly transitioning to another, allowing for the stimulation of multiple points simultaneously to induce “deqi.” (11). TCM views post-stroke motor and balance disorders as “tendon disease” and “wei syndrome,” attributed to a fundamental pathology of “imbalance of yin and yang and destitution of tendons and vessels.” Elongated needle, characterized by fewer insertions that cover a wider area of meridians, potently induces deqi. The objective of this method is to clear the meridians, harmonize qi and blood, and restore the balance of yin and yang. Studies (37) indicates that applying elongated needles along the Hegu to Houxi line can improve distal motor functions of the upper limbs and assist in the recovery of finger functions for those who have experienced a stroke. The underlying mechanism is attributed to elongated needles traversing acupoints along the conception vessel, governor vessel, and the hand-foot yang meridians, which may mitigate cerebrovascular spasms, enhance cerebral blood flow, augment blood circulation in the penumbral area surrounding the infarct, and stimulate residual brain cells, thereby restoring neurological function (38, 39). Despite Chinese clinical trials showing improvements in post-stroke motor functions, activities of daily living, and balance with elongated needle, evidence-based medicine has not yet definitively substantiated these benefits. The aim of this study is to furnish compelling evidence endorsing the application of elongated needle in addressing post-stroke limb motor and balance disorders, thereby establishing its efficacy and safety.

The study analyzed 18 original papers and revealed that the elongated needle method, either alone or in combination with other techniques, significantly enhances balance in post-stroke patients compared to conventional acupuncture, rehabilitation training, oral traditional Chinese medicine, and other therapies including electroacupuncture and acupoint application. Regarding limb motor skills, treatments involving elongated needles demonstrated considerable effectiveness. However, the small number of studies included prevents a detailed subgroup analysis of different controls on motor improvement or the establishment of an optimal elongated needle treatment protocol. In terms of daily living abilities, elongated needle, both individually and combined with other methods, was found superior to solo conventional acupuncture, rehabilitation training, and their combination. Nevertheless, further clinical research is necessary to establish conclusive evidence comparing the efficacy of elongated needle acupuncture with other interventions such as electroacupuncture or acupoint application. Different scales for assessing Activities of Daily Living (ADL) in stroke patients tend to increase heterogeneity; the Barthel Index (BI) and the Modified Barthel Index (MBI) are commonly used, although the BI may not accurately reflect the disability level in patients with higher functionality due to a “ceiling effect.” Therefore, the MBI, an adaptation of the BI, is recommended for future research (40, 41). Regarding safety, most studies reported no adverse reactions; however, three mild reactions occurred in two studies, all related to acupuncture and resolved with appropriate treatment.

This study also has several limitations: (1) The included studies were exclusively retrieved from Chinese-language databases, and no relevant clinical reports were identified in English-language repositories, resulting in a linguistically restricted evidence base. (2) The sample size in the included studies is relatively small, lacks estimation, and blinding of researchers and subjects is challenging due to the specificity of the elongated needle, resulting in a lower methodological quality of the included literature. (3)The findings demonstrated substantial heterogeneity. While sensitivity analyses and subgroup analyses identified potential contributing factors, the precise origins of residual heterogeneity remained undetermined. This may be attributed to variations in acupoint selection protocols, needling depth, needle retention duration, or baseline stroke severity. Regrettably, the limited number of included studies, combined with insufficient reporting of elongated needle intervention details (e.g., acupoint, needling depth, needle retention duration) and baseline stroke severity data in most trials, precluded robust analytical approaches to validate these potential sources of heterogeneity.(4) Syndrome differentiation and treatment (Bianzheng Lunzhi) is a defining feature of traditional Chinese medicine clinical practice. However, most included studies failed to report stroke subtypes (e.g., ischemic/hemorrhagic) or specify TCM syndrome patterns (e.g., wind-phlegm obstruction, qi deficiency). Consequently, we were unable to analyze whether elongated needle exerts differential therapeutic effects across distinct TCM syndrome classifications or stroke pathophysiological subtypes. To address these limitations, the following recommendations are proposed: (1) Establish standardized intervention protocols requiring explicit documentation of needle specifications (gauge/length), acupoint selection rationale, and needle retention duration. Future trials should adopt multicenter designs with larger sample sizes to enhance statistical power. (2) Implement more rigorous methodological designs, particularly regarding randomization procedures, blinding implementation, and allocation concealment, to minimize potential bias risks. (3) Subsequent RCTs should incorporate TCM syndrome differentiation, employing personalized acupoint selection strategies tailored to specific stroke subtypes and TCM syndrome patterns.

In conclusion, the use of elongated needles has been shown to improve balance and motor functions in post-stroke patients, enhance their capacity for daily activities, and has not been associated with significant adverse effects. Considering the constraints of this study, further rigorous, large-sample, multicenter clinical RCTs that adhere to international standards are essential to conclusively determine the clinical benefits of this acupuncture method, and to evaluate and contrast its efficacy, benefits, and potential drawbacks with other therapeutic options.

## Data Availability

The original contributions presented in the study are included in the article/supplementary material, further inquiries can be directed to the corresponding author.
